# Genome sequence analysis of *Zooshikella ganghwensis* strain VG4 and its potential for the synthesis of antimicrobial metabolites

**DOI:** 10.1016/j.btre.2018.e00278

**Published:** 2018-08-24

**Authors:** Zahid ur Rehman, Intikhab Alam, Allan Anthony Kamau, Vladimir B. Bajic, TorOve Leiknes

**Affiliations:** a Water Desalination and Reuse Center (WDRC), Saudi Arabia; b Computational Bioscience Research Centre (CBRC), Biological & Environmental Science & Engineering Division (BESE), King Abdullah University of Science and Technology (KAUST), Thuwal 23955-6900, Saudi Arabia

**Keywords:** Bioactive secondary metabolites, Nonribosomal peptides, Polyketides, PK, NRP, *Zooshikella ganghwensis* genome

## Abstract

With antimicrobial resistance on the rise, the discovery of new compounds with novel structural scaffolds exhibiting antimicrobial properties has become an important area of research. Such compounds can serve as starting points for the development of new antimicrobials. In this report, we present the draft genome sequence of the *Zooshikella ganghwensis* strain VG4, isolated from Red Sea sediments, that produces metabolites with antimicrobial properties. A genomic analysis reveals that it carries at least five gene clusters that have the potential to direct biosynthesis of bioactive secondary metabolites such as polyketides and nonribosomal peptides. By using *in-silico* approaches, we predict the structure of these metabolites.

## Highlights

• Draft genome sequence of *Z. ganghwensis* VG4 is reported.
• Culture supernatant of *Z. ganghwensis* VG4 exhibit antimicrobial properties.
• A total of 7634 genes are identified out of which 74% were annotated.
• *Z. ganghwensis* VG4 has genetic potential to synthesize bioactive secondary metabolites, such as, polyketides and nonribosomal peptides.

## 1 Introduction

The emergence and spread of resistance against known antimicrobials has renewed interest in the discovery of microbial natural products with antimicrobial properties. Recent studies have revealed that microbes found in the Red Sea can produce a variety of antimicrobial compounds [[1], [2], [3], [4]]. The sequencing of microbial genomes has revealed the immense genetic potential of microbes to synthesize bioactive secondary metabolites [5]; however, the vast majority of secondary metabolites has remained unidentified [6].

**Fig. 1:**
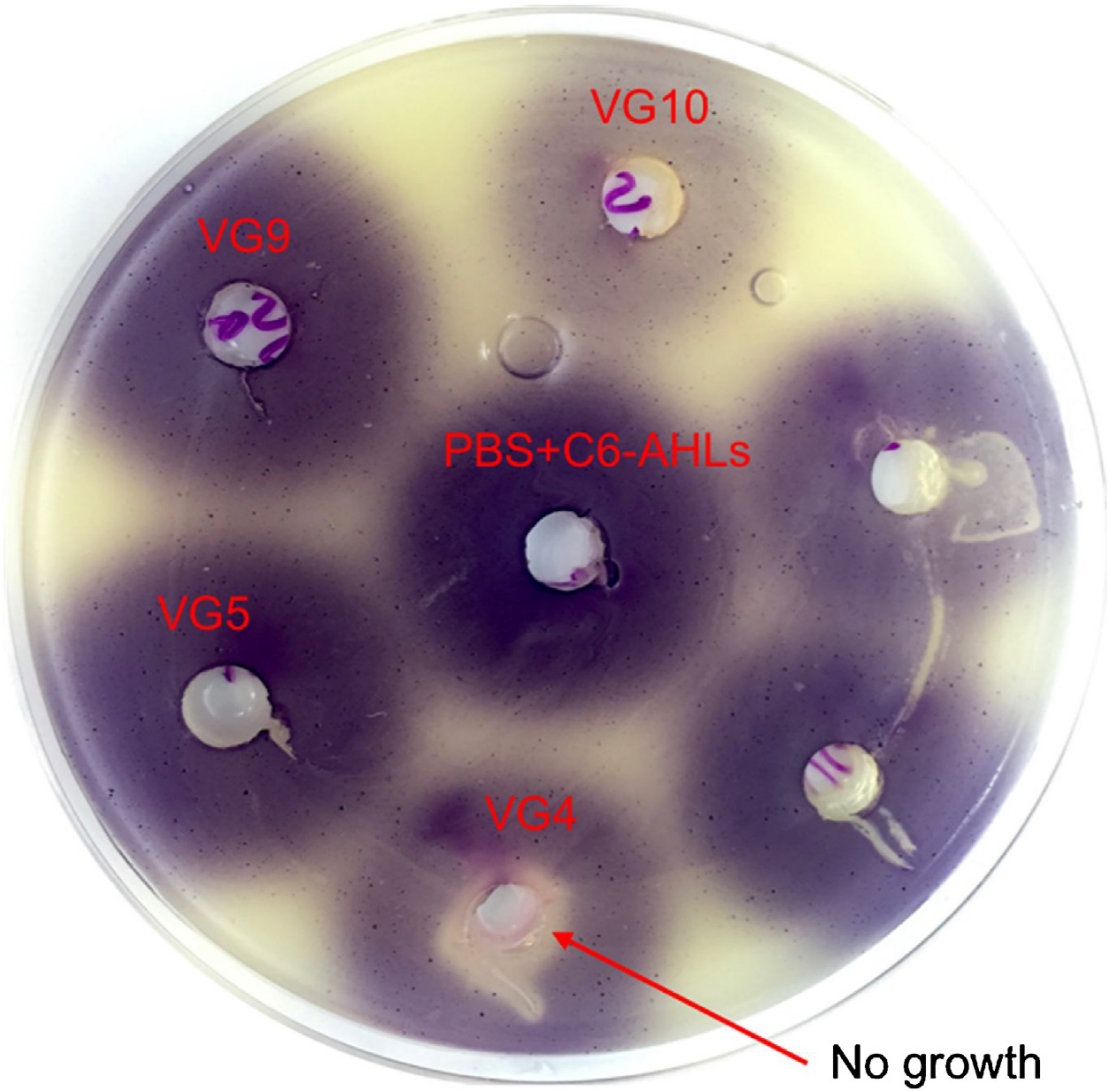
*Z. ganghwensis* strain VG4 exhibiting antimicrobial activity. A clear halo (diameter = 11 ± 1 mm including well) around VG4, shown by the arrow, showed killing of *C. violaceum*. However, *C. violaceum* was able to grow at some distance from the well, as indicated by the purple color. Other bacterial isolates (VG5, VG9, VG10) and a negative control (PBS + C6-AHLs) did not inhibit the growth of *C. violaceum*. This is a representative figure of two independent replicates.

In a recent study, we isolated bacteria, from the Red Sea sediments, in the vicinity of seagrass, and tested their ability to degrade Acyl Homoserine Lactone (AHL) molecules [7]. While doing the initial screening, we observed that the culture supernatant of one isolate could kill the biosensor strain *Chromobacter violaceum* CV026 used in the assay (Fig. 1). We hypothesized that this isolate produced secondary metabolites with antimicrobial properties. Therefore, we sequenced the genome of this isolate in order to investigate the genetic potential of this bacterium to synthesize such metabolites. The 16S-rRNA gene sequence showed a high homology (99% identity) to the *Z. ganghwensis* strain JC2044, which was isolated from sediments samples from Getbol in Korea [8]. Similarly, to other *Zooshikella* isolates, this isolate also produced a red pigment that gave a red color to the colony. The red pigment was identified as Prodigiosin, which has shown anticancer and antimicrobial properties [9,10].

## 2 Strain isolation and QQ assay

Red Sea sediments were collected at a depth of 1–2 m, from the coastal area 12 km North of Thuwal (22.389778, 39.135556), Saudi Arabia. Sediments were acquired using a 30-cm-long acrylic cylindrical tube. Sampled sediments were stored at 30 °C, and bacteria were isolated at the earliest to avoid any negative effect due to storage. For bacterial isolation, approximately 1 *g* of sea sediments were suspended in 1 mL of 0.2-μm filtered autoclaved seawater, and vortexed. This mixture was left to stand for 1–2 min to allow the bigger particles to settle down. The supernatant was then serially diluted (10-fold), and plated on Marine Agar (MA) (HIMEDIA, India). The plates were incubated at 30 °C, for 1 week. Selected bacterial colonies were further sub-cultured onto fresh agar plates. Single colonies were subsequently streaked twice to obtain pure cultures. Quorum-quenching assay was conducted as described previously [7]. Briefly, the isolates were grown in 0.5 mL of Marine broth and incubated at 30 °C with shaking. C6-AHLs were added to this bacterial culture to reach a final concentration of 10 μM and further incubated for 24 h at 30 °C with shaking. The bacterial cultures were centrifuged to pellet the cells, and the remaining C6-AHLs in the culture supernatant were detected by adding it to the wells of the LB agar plate overlaid with *C. violaceum*. The plate was incubated further for 24 h at 37 °C A purple halo indicated an absence of QQ activity (i.e no degradation of C6-AHLs), whereas no halo indicated a degradation of C6-AHLs.

## 3 Genome sequencing and analysis

For the genome sequencing, the genomic DNA was extracted, using a DNA blood and tissue kit from Qiagen. The library for the whole genome sequencing was prepared by following the Pacific Biosciences (PacBio) 20-kb Template preparation protocol, also using the BluePippin Size Selection System protocol, and subsequently sequenced on PacBio RS platform. The PacBio chemistry resulted in 49,247 reads and 7.9 Gb of data.

The PacBio sequence reads were assembled, using a CANU WGS assembler [11] version 1.4 with default parameters. Assembly of the whole genome yielded 12 contigs with N50 of 5.9Mb and a total genome size of 6.6 Mbp. GC content of the genome was 41.09%. Functional annotation of this bacterium was performed using the Automatic Annotation of Microbial Genomes (AAMG) pipeline [12]. Briefly, this annotation pipeline first validated the sequence quality using prinseq [13]. The RNA prediction was then carried out using RNAmmer [14], tRNAscan-SE [15] and Infernal [16]. Open Reading Frames (ORFs were predicted using FragGenScan [17]. RNA predictions were compared with the latest NCBI’s 16S-rRNA database and EBI’s Rfam [18] database, using nucleotide BLAST. ORFs were compared to the latest version of UniProt/Trembl [19] and KEGG [20] databases. Domains and Gene Ontology assignments were performed using high- throughput Interproscan analysis [21]. Out of a total of 7634 (ORF + RNA genes) genes that were identified, 74% were annotated (Table 1). NCBI annotations of the genome are available online at URL: https://bit.ly/2w6lelI

**Table 1:** Counts of genomic features annotated through different sources.

Genome Features	Counts
ORFs	7502
rRNA	12
tRNA	60
rfamRNA	60
Uniprot	5482
KEGG	3742
COG	2906
InterPro	4076
GO	2603
Total Annotated Genes	5639
Total Unassigned Genes	1995

(http://www.cbrc.kaust.edu.sa/aamg/1487944605297_VG4_30.0_intikhab/)

## 4 Prediction of NRP and PK synthases

It has been suggested that large enzyme complexes, such as polyketide synthases and nonribosomal peptide synthetases, synthesize the majority of the bioactive natural products [22]. Different bioinformatic approaches have been developed for identifying such enzymes in the genomes, and for predicting the structures of polyketides (PK) and nonribosomal peptides (NRP) produced by these enzyme [23,24]. These bioinformatic tools search for protein domains such as thiolation, condensation, acyltransferase, and adenylation domains that are involved in the biosynthesis of natural products.

For the prediction of PK synthases and NRP synthetases, we used an open-source web application called PRISM 3 (PRediction Informatics for Secondary Metabolomes). This computational resource is a valuable tool for the prediction of gene clusters involved in the biosynthesis of bioactive secondary metabolites such as type I and type II PK and NRP and their structures [25].

**Fig. 2:**
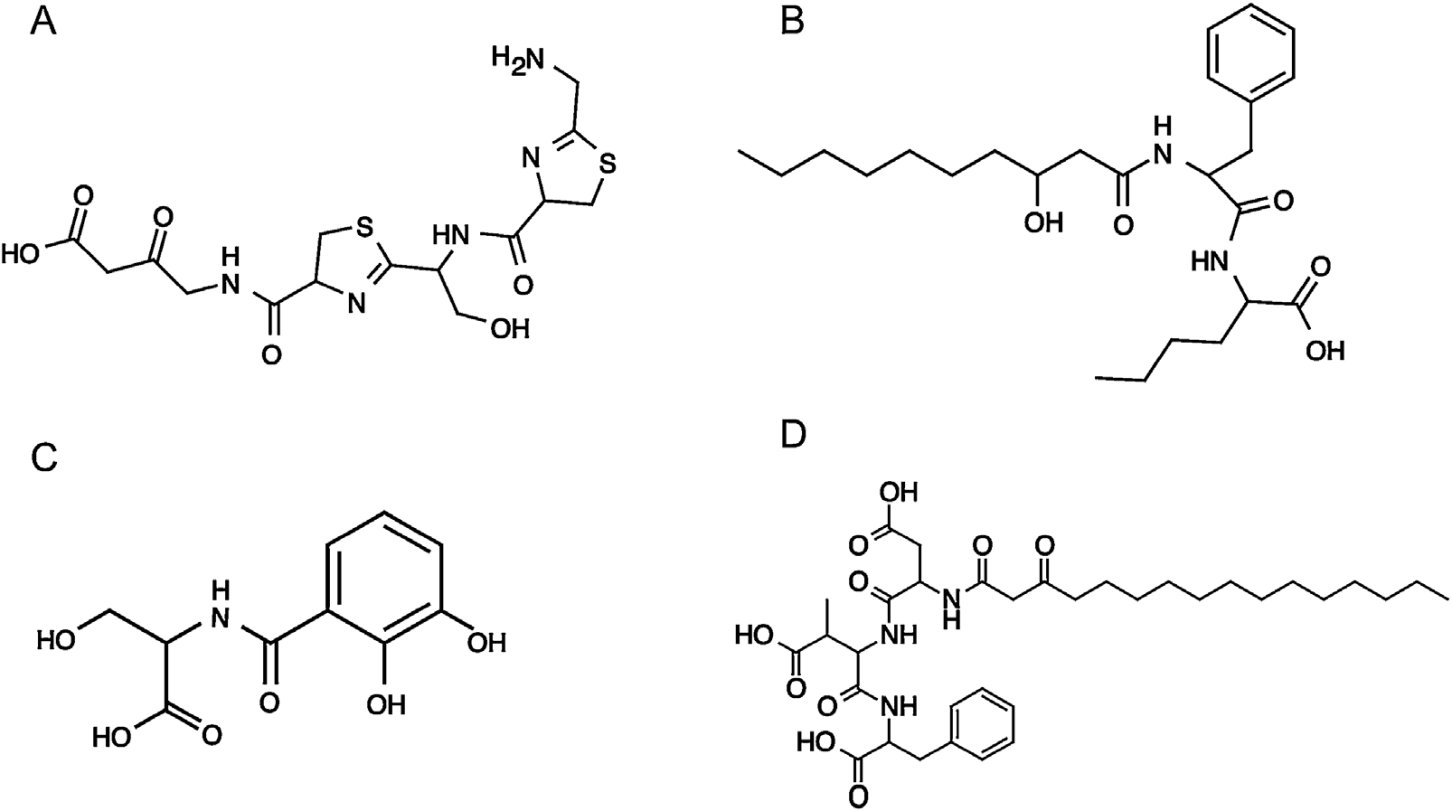
Structure of secondary metabolites predicted by PRISM. (A) Structure of NRP/PK as predicted for Cluster 1. (B), (C) Structure of NRP predicted for cluster 2 and 3 respectively and (D) structure of NRP/PK, as predicted for cluster 5. PRISM was unable to predict structure for PK synthesized by cluster 4. These metabolites were named using ChemDraw as (A) 4-(2-(1-(2-(aminomethyl)-4,5-dihydrothiazole-4-carboxamido)-2-hydroxyethyl)-4,5-dihydrothiazole-4-carboxamido)-3-oxobutanoic acid, (B) 2-(2-(3-hydroxydecanamido)-3-phenylpropanamido) hexanoic acid, (C) (2,3-dihydroxybenzoyl)serine, (D) 3-(3-carboxy-2-(3-oxohexadecanamido)propanamido)-4-((1-carboxy-2-phenylethyl)amino)-2-methyl-4-oxobutanoic acid.

An analysis of the *Z. ganghwensis* genome sequence, using PRISM, resulted in the identification of 5 gene clusters that could potentially synthesize NRP and PK (Table 2). Two of the gene clusters were capable of synthesizing both NRP and PK. It is not clear if such gene clusters can produce both PK and NRP secondary metabolites, or a molecule that is a hybrid of both. Two gene clusters synthesized only NRP, and one gene cluster synthesized only PK (Table 2). Cluster 1 consists of four open reading frames (ORF), two of which encode the antimicrobial resistance genes, a third one, VG4_000000308, that carry five domains involved in the synthesis of NRP, and a fourth ORF, VG4_000000309, that encodes a protein containing 14 domains, involved in the biosynthesis of both NRP and PK. We found that the modular structure of these ORFs was typical to that found in NRP and PK synthases [6]. The predicted structure of the secondary metabolite produced by this cluster is presented in Fig. 2A. Predicted cluster 2 contains only one ORF, and its protein product is predicted to consist of 7 domains, involved in the production of NRP. The predicted structure of NRPs produced by this ORF is shown in Fig. 2B. Cluster 3 consists of three ORFs, and can only synthesize NRP. The predicted structure of NRPs synthesized by this cluster is shown in Fig. 2C. Cluster 4 consists of four ORFs each with one domain. This cluster is capable of synthesizing only PKs (Table 2). PRISM was unable to predict the structure of PKs synthesized by this cluster. Lastly, we found that cluster 5 contained three ORFs and that the protein product of VG4_000004243 contained 9 domains, usually involved in the biosynthesis of NRP and PK. The protein product of VG4_000004245 is predicted to contain 6 domains involved in the synthesis of NRP only (Fig. 2D). We note that AAMG annotations for PRISM detected genes are in good agreement (Table 2).

**Table 2:** ORFs predicted by PRISM, their KEGG (Kyoto Encyclopedia of genes and Genomes) orthologs and functions. ORFs predicted by PRISM were BLASTed against the protein sequences predicted for VG4 genome to obtain the ORF IDs that correspond to our annotations (link given above).

Clusters	Metabolites	ORF IDs	KEGG Ortholog	AAMG Function
Cluster 1	PK/NRP	VG4_000000305	K07552, bcr	Multi drug resistance protein
		VG4_000000308	K16093, bacA	Bacitracin synthase
		VG4_000000309	K15662, mycB	Lipopeptide synthetase B
		VG4_000000311	K06158, ABCF3	ATP-binding cassette
Cluster 2	NRP	VG4_000002985	K04780, dhbF	NRP synthetase
Cluster 3	NRP	VG4_000003153	K02363, entE	2,3-dihydroxybenzoate-AMP ligase
		VG4_000003154		Isochorismatase
		VG4_000003155	K02364, entF	Enterobactin synthetase component F
		VG4_000003161	K02362, entD	Enterobactin synthetase component D
Cluster 4	PK	VG4_000003613	K02619, pabC	4-amino-4-deoxychorismate lyase
		VG4_000003614	K09458, fabF	3-oxoacyl-[acyl-carrier-protein] synthase II
		VG4_000003615	K02078, acpP	Acyl carrier protein
		VG4_000003617	K00645, fabD	S-malonyltransferase
Cluster 5	PK/NRP	VG4_000004243	K16129, mcyE	Microcystin synthetase
		VG4_000004244	K01953, asnB	Asparagine synthase
		VG4_000004245	K15667, ppsD	Lipopeptide synthetase D

## 5 Conclusions

In this study, a phenotypic and genomic analysis showed that *Z. ganghwensis* strain VG4 produced secondary metabolites with potential antimicrobial activity. This antimicrobial activity could be the result of Prodigiosin or other secondary metabolites, such as PK and NRP, that are potentially produced by this bacterium. In future studies, our goals will be to confirm the production of these metabolites and to investigate their bioactivity.

## Data deposition

The BioProject ID for this genome submission is PRJNA383317.

This Whole Genome Shotgun project was deposited at DDBJ/ENA/GenBank, under the accession number NDXW00000000. The version described in this paper is version NDXW01000000.

## Conflict of interest

The authors declare no competing financial interests.
